# Lipoid congenital adrenal hyperplasia by steroidogenic acute regulatory protein (STAR) gene mutation in an Italian infant: an uncommon cause of adrenal insufficiency

**DOI:** 10.1186/s13052-017-0371-y

**Published:** 2017-06-20

**Authors:** Carla Bizzarri, Elisa Pisaneschi, Mafalda Mucciolo, Stefania Pedicelli, Daniela Galeazzi, Antonio Novelli, Marco Cappa

**Affiliations:** 1 Unit of Endocrinology and Diabetes, “Bambino Gesù” Children’s Hospital, IRCCS, Piazza S. Onofrio 4, 00165 Rome, Italy; 2 Medical Genetics Laboratory, “Bambino Gesù” Children’s Hospital, IRCCS, Rome, Italy; 3 Pediatric Department, Azienda Unità Sanitaria Locale Umbria 2, Narni (Terni), Italy

**Keywords:** Congenital adrenal hyperplasia, Steroidogenic acute regulatory protein, Adrenal insufficiency, infant

## Abstract

**Background:**

Lipoid congenital adrenal hyperplasia (CAH) (OMIM n. 201710) is the most severe form of congenital adrenal hyperplasia. It is characterized by severe adrenal and gonadal steroidogenesis impairment due to a defect in the conversion of cholesterol to pregnenolone. Affected infants experience salt loss, but glucocorticoid and mineralocorticoid replacement therapy enables long-term survival. Classic lipoid congenital adrenal hyperplasia is relatively common in Japan and Korea but extremely rare in Caucasian populations.

**Case presentation:**

A female infant of Italian origin came to our attention in late infancy with a clinical picture of acute adrenal insufficiency.

The study of the *STAR* gene revealed two genomic variants c.562C > T and c.577C > T in compound heterozygosity. At the protein level, the two mutations determine the p.Arg188Cys variant (rs104894090) and the p.Arg193Ter variant (rs387907235), respectively. Sanger sequencing was used to confirm the identified variants and to perform familial study. The mother carried the p.Arg188Cys variant, while the father carried the p.Arg193Ter variant.

**Conclusion:**

To our knowledge this is the second case of classic lipoid congenital adrenal hyperplasia reported in the Italian population*. STAR* mutations resulting in lipoid congenital adrenal hyperplasia should be considered all over the world in the differential diagnosis of newborn babies and infants with primary adrenal insufficiency.

## Background

Lipoid congenital adrenal hyperplasia (CAH) (OMIM n. 201710) is the most severe form of CAH. It is characterized by severe adrenal and gonadal steroidogenesis impairment due to a defect in the conversion of cholesterol to pregnenolone [1]. Affected infants experience salt loss from impaired mineralocorticoid and glucocorticoid synthesis, but hormonal replacement therapy enables long-term survival [2]. Steroidogenesis begins with cellular internalization of low-density lipoprotein (LDL) particles. The means by which cholesterol is directed to steroidogenic mitochondria is not fully understood [1, 3, 4]. Once cholesterol reaches the outer mitochondrial membrane, the steroidogenic acute regulatory protein (STAR) regulates its delivery to the inner mitochondrial membrane, where it becomes the substrate for the cholesterol side-chain cleavage enzyme, P450scc [1, 3, 4]. The human *STAR* gene is located on chromosome 8p11.2 and consists of seven exons [1, 5]. It is translated into a 285-amino acid protein including a mitochondrial target sequence (N-terminal), which drives STAR to the inner mitochondrial membrane and a cholesterol binding site (C-terminal). Children affected by lipoid CAH have low but measurable levels of steroid hormones at birth, due to low levels of STAR-independent steroidogenesis [3, 4], The demonstration of STAR-independent steroidogenesis led to the formulation of the two-hit model of lipoid CAH [3, 4]. The first hit is the mutation in the *STAR* gene, ablating STAR-dependent steroidogenesis, but permitting STAR-independent steroidogenesis [4]. This enables normal placental steroidogenesis and term gestation, and explains the low detectable levels of steroid hormones in newborns with lipoid CAH and why infants with untreated lipoid CAH can survive without treatment for a few months [4]. These steroid hormone levels are too low to suppress secretion of adrenocorticotropic hormone (ACTH), gonadotropins, and angiotensin II. These tropic hormones stimulate cellular uptake of LDL cholesterol and increase production of cholesterol from acetate, resulting in the accumulation of cholesterol esters in lipid droplets, which finally destroy cells either by progressive enlargement or by a chemical action of cholesterol oxidation products [4]. This second hit disrupts the low levels of STAR independent steroidogenesis, leading to undetectable steroid levels in older infants [4]. Unlike the testes and adrenal glands, the ovaries produce steroids at the onset of puberty [4, 6]. Therefore, the ovaries of 46,XX females with lipoid CAH do not receive the second hit until the onset of puberty, when luteinizing hormone stimulates low-levels of STAR-independent steroidogenesis. Affected girls have spontaneous puberty. Each month a follicle is recruited by gonadotropins [6, 7], but stimulation quickly results in cholesterol accumulation in these cells (the second hit in lipoid CAH), so ovulation and the luteal phase with progesterone secretion do not occur. Follicles that are not recruited constitute a reservoir of steroidogenic cells undamaged by the second hit. A new undamaged follicle is recruited at each cycle, and estrogens are produced leading to cyclic uterine withdrawal bleeding that resembles a normal menstruation, but there is no progesterone, so these cycles are anovulatory [6]. In vitro studies revealed that STAR protein lacking the N-terminal targeting sequence is still able to stimulate steroidogenesis, whereas mutations in the C-terminal region lead to severely diminished or absent function [6]. Lipoid CAH is relatively common in Japan and Korea. To date, 48 different mutations in the *STAR* gene have been reported in various ethnic groups (http://www.hgmd.org). The genetic defect in the *STAR* gene in Japanese and Korean patients is highly homogeneous, probably reflecting a founder effect. Approximately 65–70% of affected Japanese alleles and virtually all affected Korean alleles carry the mutation Q258X. The Q258X carrier frequency in these countries is about 1 in 300 [7] so that 1/250,000 to 1/300,000 newborns is affected. Other genetic clusters were found among Palestinian Arabs, carrying the mutation R182L [4], in eastern Saudi Arabia, carrying the mutation R182H [7], and in Switzerland, carrying the mutation L260P [7]. Most disease-causing *STAR* mutations are located in the C-terminal region between exon 5 and 7, encoding for STAR related lipid transfer domain [4], they do not have measurable activity and cause classic lipoid CAH when homozygous or in compound heterozygosity with mutations of similar activity. A milder form of lipoid CAH, defined as “non-classic,” is related to mutations retaining 10–25% of normal STAR activity [7]. These patients typically experience adrenal insufficiency after infancy, mineralocorticoid secretion is minimally affected and the 46,XY individuals may masculinize normally [8–10].

To our knowledge, only one case of classic lipoid CAH has already been described in the Italian population [11]. We report a new Italian case of classic lipoid CAH with onset during late infancy, and a clinical presentation of intermediate severity.

### Patient report

A female infant was born by caesarean section at the 33rd week of gestation due to maternal eclampsia and rupture of membranes, from a twin pregnancy induced by in vitro fertilization and embryo transfer. Both parents were Caucasian of Italian ancestry, healthy and non-consanguineous. The twin brother was in apparent good health. Birth weight was 1960 g (0.17 SDS), length 43 cm (−0.06 SDS). Apgar scores at the 1st and the 5th min of life were 9 and 10, respectively. There was no family history of disorders of sex development (DSD) or sudden death. The infant did not have neonatal hypoglycemia or respiratory distress. Newborn screening for congenital adrenal hyperplasia was not performed. She had a normal weight gain until 7 months of age, when she was admitted to a peripheral hospital for viral gastroenteritis, with significant weight loss, dehydration and mild hyponatremia (Na 128 mEq/L). One month later, she presented a new episode of gastroenteritis. Laboratory data at this time showed severe hyponatremia (Na 110 mEq/L), hypochloraemia (Cl 74 mEq/L) and hyperkalemia (K 6.3 mEq/L). Mild skin hyper-pigmentation was evident on the face, hands, elbows and knees. Blood glucose levels were moderately low during both these episodes (61 mg/dl and 63 mg/dl, respectively). Vital signs detected during both the episodes were in the normal ranges for age (blood pressure 96/48–98/45 mmHg, heart rate 124–130 beats/min, and respiratory rate 30–31 breaths/min). Intravenous therapy with high doses of hydrocortisone was started together with rehydration, until stabilization of clinical conditions. After that, the child was referred to our tertiary health care center, with the suspicion of classic CAH.

At admission in our Unit weight was 5.4 kg (< −2 SDS), length 65 cm (−1.0 SDS). Table 1 shows blood steroid profile at admission. Commercial kits were used for the estimation of 17*-*hydroxyprogesterone (RIA, ICN-Pharmaceutical Inc.) and plasma renin (RIA, Sanofi-Pasteur). Delta-4-androstenedione, cortisol and testosterone levels were measured by chemiluminescence using Siemens IMMULITE-2000 XP analyzer (Siemens Healthcare Diagnostic Products, Milan, Italy). ACTH concentrations were determined by immonoradiometric assay (ACTH IRMA, Nichols Institute Diagnostics), with a sensitivity of 1 pg/mL. All the reported reference ranges are based on sex and age. At ultrasound, the child showed normal uterus and ovaries, while adrenal glands appeared mildly enlarged. After the resolution of dehydration and salt losing, oral replacement therapy with hydrocortisone (16 mg/m^2^ of body-surface area/day) and fludrocortisone (0.1 mg/day) was started, in conjunction with oral sodium chloride supplementation (5 mEq for five times a day). The study of karyotype showed a normal 46,XX female.

**Table 1:** Blood steroid profile at admission

	Patient’s blood level	Reference range
17-hydroxyprogesterone (ng/mL)	<0.1	0.1–1.78
Androstenedione (ng/dL)	1.9	5–45
Testosterone (ng/dL)	<10	<10
Cortisol (mcg/dL)	2.91	0.6–19.8
ACTH (pg/ml)	1250	96–135
Renin (mcUI/mL)	450–500	4–89
Aldosterone (pg/ml)	25–155	320–1300

The glucocorticoid and mineralocorticoid deficiency, associated with low levels of testosterone and androgen precursors, without evidence of DSD, suggested an abnormality of a gene affecting adrenal function. Sequencing of the ACTH receptor (*MC2R*), DAX1 (AHC, *NR0B1*), and SF1 (steroidogenic factor 1, *NR5A1*) genes did not show any variants.

**Fig. 1:**
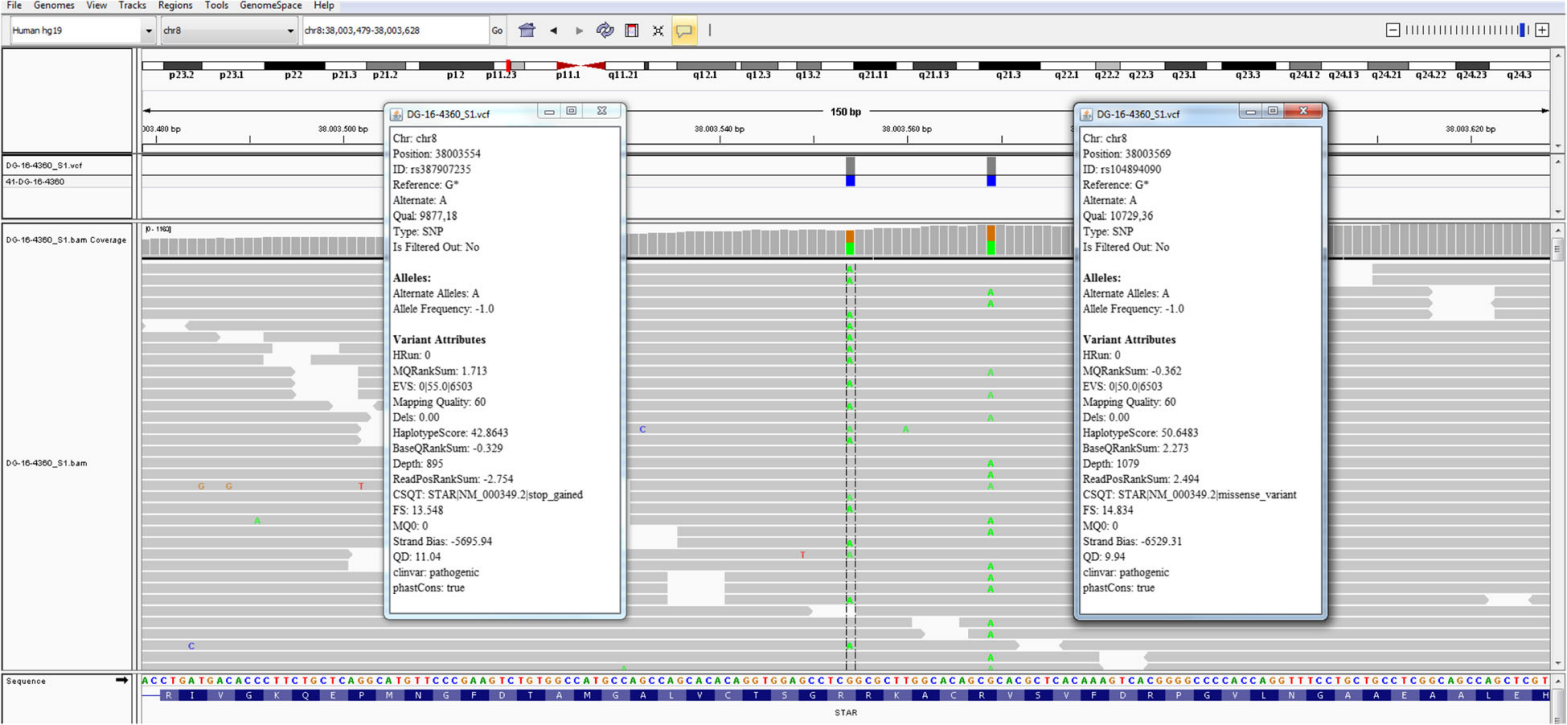
Next Generation Sequencing (NGS) analysis: variant visualization on IGV (Integrative Genome Viewer) and variant annotation (Variant Studio)

**Fig. 2:**
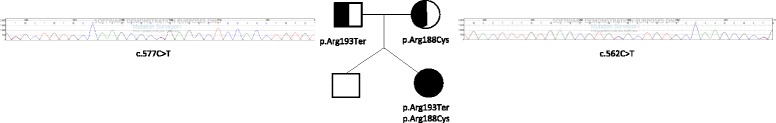
Familial Study: Sanger sequencing to confirm variants and parental analysis

Sequencing the *STAR* gene revealed two genomic variants c.562C > T and c.577C > T in compound heterozygosity. At the protein level the two mutations determine the p.Arg188Cys variant (rs104894090) and p.Arg193Ter variant (rs387907235), respectively. The study was performed by amplification and high-throughput sequencing with Nextera Rapid Capture Custom Enrichment Kit (Illumina) using customized panel and analyzed with the NextSeq550® sequencing platform (Illumina, San Diego, CA), with sensitivity and analytical specificity >99% (Fig. 1). *STAR* gene is in the panel of targeted genes for the analysis of DSD. Sanger sequencing was used to confirm the identified variants and to perform familial study, this is important to determine variant segregation (Fig. 2). The mother carried the p.Arg188Cys variant, while the father carried the p.Arg193Ter variant. In the SNP scientific (Single Nucleotide Polymorphism) databases the variants have a low frequency. Pathogenicity prediction software (Sift, PolyPhen) defines the variants as deleterious or probably damaging. Their pathogenicity is confirmed in the reference literature [12–14].

## Discussion

*STAR* gene mutations have now been described in at least 190 patients with lipoid CAH, including 100 patients available in Japanese literature. In most studies the reported patients were siblings, or they were born from consanguineous marriages. The present study is the first one to identify the *STAR* c.562C > T and c.577C > T mutations in a patient of Italian ancestry.

The p.Arg188Cys variant was previously reported in two 46,XY brothers with normal male genitalia and descended testes, belonging to a consanguineous Pakistani family [12]. The older boy developed hyperpigmentation at 1.5 years; investigation at 2.2 years of age revealed compensated primary adrenal failure with low basal cortisol, elevated ACTH, mildly elevated renin activity and no cortisol response to ACTH stimulation. The younger brother also became progressively pigmented. At 2.8 years of age, his cortisol, aldosterone, and electrolytes were normal, but ACTH and renin activity were elevated, and cortisol did not respond to ACTH. Functional analysis, performed on non-steroidogenic cells transfected with vectors expressing both the cholesterol side-chain cleavage enzyme and the STAR protein, demonstrated that Arg188Cys mutants retained 13.6% of wild-type activity [12].

The second mutation of our patient is the p.Arg193Ter variant, a nonsense mutation inserting a premature stop codon that truncates the STAR protein at 193 amino acids. Given that the deletion of 10 amino acids from the C-terminal destroys approximately 50% of its activity [7], it is expected that this mutation severely impairs or abolishes pregnenolone synthesis. This mutation has been recently described in a 2.5 month old Norwegian 46,XY infant presenting with adrenal salt wasting crisis and normal female external genitalia [13]. The baby showed also the *STAR* p.N148 K mutation in exon 4, which had been already described in an Italian patient [11]. Genomic DNA analysis of both parents revealed the absence of mutations, suggesting that both mutations were de novo. Functional analysis showed that *STAR* p.N148 K is a loss of function mutation, resulting in absent protein activity [8, 9]. Our patient was a compound heterozygous for a missense mutation (p.Arg188Cys) and a stop mutation (p.Arg193Ter) and she showed an intermediate phenotype without neonatal adrenal crisis. Clinical presentation occurred around six months of life, during an intercurrent illness unmasking for the first time the probably chronic, but until then compensated, salt losing. Surprisingly, no overt hypoglycemia related to the glucocorticoid insufficiency was evident. Baker et al. [12] reported that homozygous Arg188Cys mutations of the *STAR* gene cause non-classic presentation of lipoid CAH. In this case functional studies were not performed, but based on previous reports, we can suppose that the later onset and the relatively milder presentation are related to the residual STAR functional activity (around 13%) of the Arg188Cys mutant.

A recently published case series of primary adrenocortical insufficiency reported 5 Caucasian patients carrying the homozygous p.R188C mutation. All patients belonged to an isolated Canadian population of European ancestry, and a founder effect was supposed [10]. It has been assumed that de novo mutations could be responsible for the isolated cases of lipoid CAH reported in countries where the disease is extremely uncommon [13]. In our case, both the mutations were inherited and no parental consanguinity was evident, suggesting the potential presence of *STAR* heterozygous mutations in the background population, albeit with low frequency. *STAR* mutations may be more prevalent in some geographical areas, but not necessarily restricted to those regions. Genetic 46,XY males with female external genitalia are more likely to be diagnosed, because the lack of Mullerian ducts is easily documented by ultrasound, suggesting the diagnosis of DSD. Genetic 46,XX females have the same prevalence of this autosomal recessive disorder. Even today, based on the absence of a DSD, genetic females may not be analyzed for defects involving both adrenal and gonadal steroidogenesis and they may be considered affected by idiopathic adrenal insufficiency [14].

## Conclusion

In the future, *StAR* mutations (both inherited and de novo mutations) resulting in classic lipoid CAH should be considered all over the world in the differential diagnosis of newborn babies and infants with primary adrenal insufficiency.

## Abbreviations

ACTH: Adrenocorticotropic hormone
CAH: Congenital adrenal hyperplasia
DSD: Disorder of sex development
STAR: Steroidogenic acute regulatory protein

## References

[1] Miller WL (1997). Congenital lipoid adrenal hyperplasia: the human gene knockout of the steroidogenic acute regulatory protein. J Mol Endocrinol.

[2] Hauffa BP, Miller WL, Grumbach MM, Conte FA, Kaplan SL (1985). Congenital adrenal hyperplasia due to deficient cholesterol side-chain cleavage activity (20,22 desmolase) in a patient treated for 18 years. Clin Endocrinol.

[3] Lin D, Sugawara T, Strauss JF, Clark BJ, Stocco DM, Saenger P (1995). Role of steroidogenic acute regulatory protein in adrenal and gonadal steroidogenesis. Science.

[4] Bose HS, Sugawara T, Strauss JF, Miller WL (1996). The pathophysiology and genetics of congenital lipoid adrenal hyperplasia. N Engl J Med.

[5] Sugawara T, Lin D, Holt JA, Martin KO, Javitt NB, Miller WL (1995). Structure of the human steroidogenic acute regulatory protein (StAR) gene: StAR stimulates mitochondrial cholesterol 27-hydroxylase activity. Biochemistry.

[6] Miller WL (2002). Androgen biosynthesis from cholesterol to DHEA. Mol Cell Endocrinol.

[7] Miller WL, Bose HS (2011). Early steps in steroidogenesis: intracellular cholesterol trafficking. J Lipid Res.

[8] Metherell LA, Naville D, Halaby G, Begeot M, Huebner A, Nürnberg G (2009). Non classic lipoid congenital adrenal hyperplasia masquerading as familial glucocorticoid deficiency. J Clin Endocrinol Metab.

[9] Miller WL, Auchus RJ (2011). The molecular biology, biochemistry, and physiology of human steroidogenesis and its disorders. Endocr Rev.

[10] Tsai SL, Green J, Metherell LA, Curtis F, Fernandez B, Healey A (2016). Primary Adrenocortical Insufficiency Case Series: Genetic Etiologies More Common than Expected. Horm Res Paediatr.

[11] Bens S, Mohn A, Yüksel B, Kulle AE, Michalek M, Chiarelli F (2010). Congenital lipoid adrenal hyperplasia: functional characterization of three novel mutations in the STAR gene. J Clin Endocrinol Metab.

[12] Baker BY, Lin L, Kim CJ, Raza J, Smith CP, Miller WL (2006). Non-classic congenital lipoid adrenal hyperplasia: a new disorder of the steroidogenic acute regulatory protein with very late presentation and normal male genitalia. J Clin Endocrinol Metab.

[13] Kaur J, Casas L, Bose HS (2016). Lipoid congenital adrenal hyperplasia due to STAR mutations in a Caucasian patient. Endocrinol Diabetes Metab Case Rep.

[14] Miller WL (2017). Disorders in the initial steps of steroid hormone synthesis. J Steroid Biochem Mol Biol.

